# GlioPredictor: a deep learning model for identification of high-risk adult *IDH*-mutant glioma towards adjuvant treatment planning

**DOI:** 10.1038/s41598-024-51765-6

**Published:** 2024-01-25

**Authors:** Shuhua Zheng, Nikhil Rammohan, Timothy Sita, P. Troy Teo, Yilin Wu, Maciej Lesniak, Sean Sachdev, Tarita O. Thomas

**Affiliations:** 1 Department of Radiation Oncology, Northwestern University Feinberg School of Medicine, Chicago, IL USA; 2 Department of Radiation Oncology, Washington University School of Medicine, St. Louis, MO USA; 3 Department of Mathematics, DigiPen Institute of Technology, Redmond, WA USA; 4 Department of Neurological Surgery, Northwestern University Feinberg School of Medicine, Chicago, IL USA; 5 Department of Radiation Oncology, Northwestern Medical Group, Northwestern Memorial Hospital, Northwestern University Feinberg School of Medicine, Chicago, USA

**Keywords:** Genetics, Cancer, Cancer models, Cancer screening, CNS cancer

## Abstract

Identification of isocitrate dehydrogenase (*IDH*)-mutant glioma patients at high risk of early progression is critical for radiotherapy treatment planning. Currently tools to stratify risk of early progression are lacking. We sought to identify a combination of molecular markers that could be used to identify patients who may have a greater need for adjuvant radiation therapy machine learning technology. 507 WHO Grade 2 and 3 glioma cases from The Cancer Genome Atlas, and 1309 cases from AACR GENIE v13.0 datasets were studied for genetic disparities between *IDH1*-wildtype and *IDH1*-mutant cohorts, and between different age groups. Genetic features such as mutations and copy number variations (CNVs) correlated with *IDH1* mutation status were selected as potential inputs to train artificial neural networks (ANNs) to predict *IDH1* mutation status. Grade 2 and 3 glioma cases from the Memorial Sloan Kettering dataset (n = 404) and Grade 3 glioma cases with subtotal resection (STR) from Northwestern University (NU) (n = 21) were used to further evaluate the best performing ANN model as independent datasets. *IDH1* mutation is associated with decreased CNVs of *EGFR* (21% vs. 3%), *CDKN2A* (20% vs. 6%), *PTEN* (14% vs. 1.7%), and increased percentage of mutations for *TP53* (15% vs. 63%), and *ATRX* (10% vs. 54%), which were all statistically significant (*p* < 0.001). Age > 40 was unable to identify high-risk *IDH1*-mutant with early progression. A glioma early progression risk prediction (GlioPredictor) score generated from the best performing ANN model (6/6/6/6/2/1) with 6 inputs, including CNVs of *EGFR*, *PTEN* and *CDKN2A*, mutation status of *TP53* and *ATRX*, patient’s age can predict *IDH1* mutation status with over 90% accuracy. The GlioPredictor score identified a subgroup of high-risk *IDH1*-mutant in TCGA and NU datasets with early disease progression (*p* = 0.0019, 0.0238, respectively). The GlioPredictor that integrates age at diagnosis, CNVs of *EGFR*, *CDKN2A*, *PTEN* and mutation status of *TP53*, and *ATRX* can identify a small cohort of *IDH*-mutant with high risk of early progression. The current version of GlioPredictor mainly incorporated clinically often tested genetic biomarkers. Considering complexity of clinical and genetic features that correlate with glioma progression, future derivatives of GlioPredictor incorporating more inputs can be a potential supplement for adjuvant radiotherapy patient selection of *IDH*-mutant glioma patients.

## Introduction

Isocitrate dehydrogenase (*IDH*)-mutant glioma patients are often diagnosed at a young age (median age of 36 and 38 years for WHO Grades 2/3 and Grade 4, respectively) with a median survival over 12.3 years^1–4^. Most recently, the INDIGO trial demonstrated efficacy of targeted therapy against mutant *IDH1/2* as a well-tolerated treatment option for *IDH*-mutant glioma patients^5^. The young age at diagnosis, long-term toxicity of adjuvant radiation treatment, and availability of targeted therapy pose unique challenges for physicians, particularly radiation oncologists. Identification of *IDH*-mutant glioma patients at high risk of early progression is critical for personalized radiotherapy treatment planning.

According to the 2021 WHO central nervous system (CNS) classification system, the *ATRX* (alpha-thalassemia/mental retardation, X-linked) retained and 1p/19q-codeleted group defines a WHO Grade 2 or Grade 3 1p/19q codeleted oligodendroglioma; *ATRX* lost and homozygous deletion of *CDKN2A/B* is sufficient to classify *IDH*-mutant glioma as WHO Grade 4, and those without *CDKN2A/B* deletion are WHO Grade 2 or 3 astrocytoma^6,7^. Therefore, multiple possible WHO Grades can be designated within a biomarker-defined diagnostic entity, representing a major departure from prior histology-based CNS tumor classifications and highlighting the importance of molecular biomarkers in guiding glioma treatment^6^. Molecular biomarkers currently used for *IDH*-mutant glioma classification have complex interrelationships and multiple other molecular biomarkers are emerging as potential new candidates. Therefore, a systematic selection and integration of candidate biomarkers for risk assessment of *IDH*-mutant glioma is warranted. To this end, the objective of this study is to train and validate a supervised machine-learning (ML) based algorithm to identify *IDH*-mutant glioma patient at high risk of early progression.

Supervised ML is now widely used in the medical field to produce models and classifiers from training data for automation of tasks. Artificial neural network (ANN) is a subtype of ML technology that can analyze large datasets as inputs and make predictions with the probability of accuracy as outputs^8,9^. An ANN with two or more hidden layers is often called a deep neural network (DNN), which is particularly robust in making predictions for complex situations^10,11^. Basic requirements for supervised DNN training includes identification of relevant inputs with reduced dimensionality and redundancy, as well as a set of accurately labeled training data as output values^12^. Due to the longevity and lack of accurate long-term follow-up data of *IDH*-mutant patients, disease progression information in large public datasets is often censored. We attempted to identify and train genetic and clinical features that have no direct causal relation with *IDH* status to identify *IDH*-mutant glioma patients that have similar genetic background as *IDH*-wildtype.

## Methods

### Patient selection

**Figure 1:**
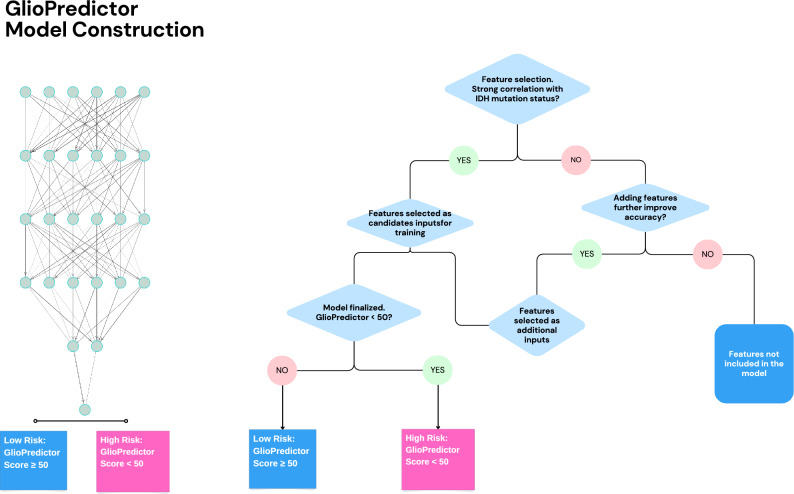
Schematic overview of model training. Left panel, illustration of the GlioPredictor structure. The neural network construction starts with identification of proper features, and trial and error in refining the inputs, model hyperparameters. New features can be added if they can further improve the performance of the model.

**Figure 2:**
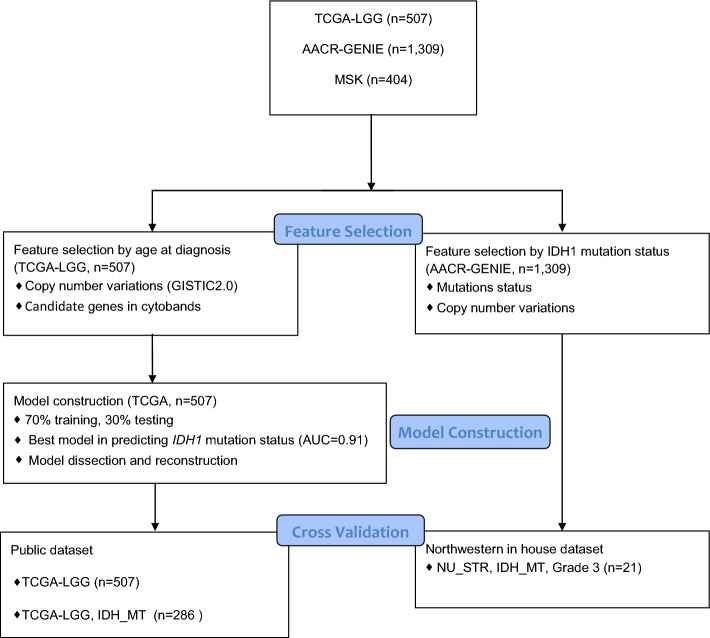
Flowchart showing the development process of GlioPredictor. *NU* Northwestern University; *STR* subtotal resection; *TCGA* The Cancer Genome Atlas; *AACR GENIE* American Associate for Cancer Research, Genomics Evidence Neoplasia Information Exchange; *MT* mutated type.

Training and validation of artificial neural networks (ANNs) were carried out using WHO Grade 2 and Grade 3 cases from The Cancer Genome Atlas dataset. Copy number variations (CNVs) of genes such as *PTEN*, *EGFR*, *CDKN2A*, and mutation status of genes such as *IDH1*, *TP53,* and *ATRX*, clinical data including age, gender, progression-free interval (PFI), overall survival (OS) days, as well as histological classifications of the TCGA cases were derived from the UCSC xena platform (https://xenabrowser.net/) (Fig. 1). CNVs and RNA-Seq raw data were processed using GISTIC2.0 and Log2(norm_count + 1) algorithms, respectively. 1309 and 404 Grade 2 and Grade 3 glioma cases from AACR GENIE v13.0 and Memorial Sloan Kettering (MSK) datasets, respectively, were derived from publicly assessable cBioportal (https://www.cbioportal.org/). This retrospective study followed the STROBE reporting guideline for publicly available datasets including TCGA, and MSK datasets included in the cBioportal. Data downloaded from a publicly available cBioPortal database does not require ethical approval. All patients whose samples were used in this analysis signed informed consent (https://docs.cbioportal.org/user-guide/faq/). *IDH1*-mutant WHO Grade 3 cases with subtotal resection (STR) who received adjuvant concurrent chemoRT were derived from Northwestern University (NU) (n = 21). Patient data was accessed with the approval of the Institutional Review Board (Study number STU00213078, August 2020) and was performed in accordance with the 45 Code of Federal Regulations Part 46 (45 CFR 46), Protection of Human Subjects (https://irb.northwestern.edu/about/, irb@northwestern.edu ). The workflow of datasets used for construction and validation of the model was illustrated in Fig. 2.

### Genomic alterations and genetic mutations

Comparison and alignment of WHO Grade 2 and Grade 3 cases from TCGA dataset (n = 516) of most frequently altered chromosome cytobands were conducted in Firebrowse (http://firebrowse.org/). Cases were first aligned by patients' age at glioma diagnosis. Corresponding cases with mutated genes were indicated and types of mutations, including frameshift, splice site, missense, inframe, and synonymous mutations, were color-coded. The most frequently mutated genes were listed. Copy number gain and loss are also listed based on the frequency of alterations. The prevalence of genetic mutations, CNVs of WHO Grade 2 and Grade 3 patients were studied at different age groups (18–40, 40–60, > 60) for MSK datasets (n = 279). Age at diagnosis less than 18 were included in the 18–40 subgroup. Genetic markers from 1309 glioma patients of AACR GENIE v13.0 dataset were sub-grouped into *IDH1*-mutant (*IDH1*_MT) and -wildtype (*IDH1*_WT) and aligned based on CNVs status of *EGFR*, *CDKN2A*, and *PTEN*, as well as mutation status of *TP53* and *ATRX*.

### Data preprocessing

Input data preprocessing was carried out in the Jupyter Notebook using Python programming language. The TCGA cases that had missing data on any input parameters were dropped. In the binary output, ‘0’ stands for *IDH1* mutated, ‘1’ stands for *IDH1*_WT. Genetic inputs with missense mutation, and truncating mutations including nonsense, frameshift, nonstart, nonstop, and splice mutations were considered positive and were assigned ‘0’, wildtype inputs were assigned ‘1’. Cases were randomly assigned to the training set, and 30% were assigned to the validation set. Inputs were selected and tested based on its variation prevalence in glioma (> 20%) and features with a correlation coefficient of > 0.2 or < − 0.2 were considered to be positively or negatively correlated.

### ANN model construction and performance assessment

**Figure 3:**
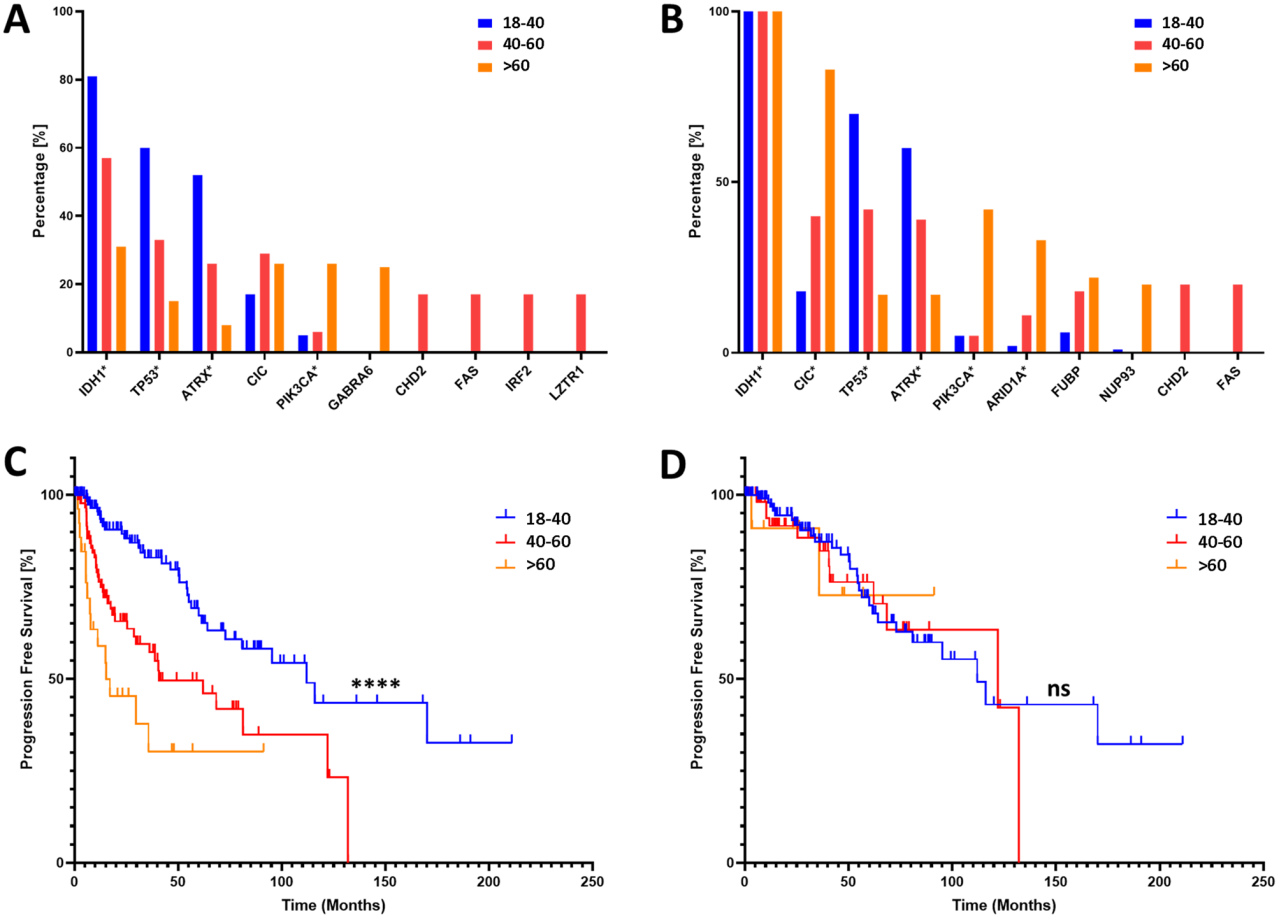
Assessment of genetics and survival of glioma patients at different age groups. (**A**) WHO Grade 2 and 3 (n = 279) patients from MSK were grouped into different age groups (18–40, 40–60, > 60) based on the age at initial diagnosis of the disease. Genes with highest frequency in any subgroup were presented. (**B**) Similarly, *IDH1* mutated glioma patients from MSK were grouped into different age groups (18–40, 40–60, > 60) based on the age at initial diagnosis of the disease. (**C**) Progression free survival (PFS) of glioma patients (n = 250) at different age groups of 18–40 (n = 131), 40–60 (n = 91), > 60 (n = 28) from MSK datasets with primary samples available (*p* < 0.0001). (**D**) PFS of WHO Grade 2 and 3 glioma *IDH1*-MT patients at different age groups of 18–40 (n = 113), 40–60 (n = 57), > 60 (n = 12) from MSK dataset with primary samples available (*p* = 0.89). ****p < 0.0001; *ns* statistically non-significant.

The model Sequential was imported from the Keras Python library. Briefly, the argument Dense was deployed for each layer with activation function relu for all the hidden layers. Since it is a binary classification task, sigmoid and adam were chosen as the activation function and optimizer, respectively, for the output layer. The loss function was fetched with the ‘binary_crossentropy’ command. The ‘early_stop’ and accuracy functions were deployed to prevent overfitting and evaluate models’ performance, respectively. Accuracy and loss function for both the training set and the validation set were plotted for each epoch. Figure 3C is a schematic overview of the architecture of the ANN (6/6/6/6/1). The best performing ANN was named as GlioPredictor for prediction of glioma early progression, with weights and biases derived from Python and reconstructed in Microsoft Excel^®^. A GlioPredictor score was calculated as 100 minus the integral numbers of sigmoid activation value that was multiplied by one hundred:

$${\text{GlioPredictor}}\;{\text{Score}} = (100 - {\text{INT}}(100*1/(1 + e^{( - x)} )).$$

### Statistical analysis

Progression-free survival (PFS) analyses were carried out using the GraphPad Prism version 8.0. Patients at risk at major time points were listed. Log-rank analysis was used to generate survival curves. Violin plot and one-way ANOVA analyses of GlioPredictor score for TCGA datasets were also carried out using GraphPad Prism. Python 3.9.0 was used for data analysis and model construction. Correlation analysis was performed using the ‘corr()’ command, which corresponds to pairwise Pearson analysis. ROC curve and AUC score analyses were conducted using ‘roc_curve’ and ‘roc_auc_score’ functions derived from the sklearn package. Univariate and multivariate analyses were carried out using IBM^®^ SPSS^®^. All statistical tests were 2-sided, and *p*-values smaller than 0.05 were considered statistically significant.

## Results

### Different age at *IDH*-mutant glioma diagnosis reflects unique genetic features

Age > 40 years at diagnosis was the criterion adopted by multiple guidelines in risk stratification for glioma patients^13,14^. We first tested potential genetic discrepancies of WHO Grade 2 and 3 diffuse glioma cases diagnosed at different ages. Progression free survival (PFS) data were derived from the Memorial Sloan Kettering (MSK, n = 250) dataset. Patients were subgrouped based on age at disease diagnosis (18–40, 41–60, > 60) (Fig. 3). We found 81% of patients aged 20–40 have *IDH1* mutation, compared to 31% in age > 60 (Fig. 3A). Younger glioma patients have significantly better PFS rates (Fig. 3C, p < **0.001**). However, for *IDH1*-mutant (*IDH1*_MT) glioma cases (n = 182), age at disease diagnosis had no significant impact on PFS rates (Fig. 3D, p = **0.89**). This finding is also true in the independent TCGA dataset (Supplementary Fig. 1). Genes with the most prevalent mutations at different age groups were presented in both *IDH1*_WT and *IDH1*_MT glioma (Fig. 3A,B). We found, regardless of *IDH1* mutation status, younger glioma patients have statistically significant higher prevalence of *P53* or *ATRX* mutations, and lower *PICK3CA* mutations (*p* < 0.05, Fig. 3A,B). These data indicated that age at glioma diagnosis reflects a unique genetic background and using age alone cannot predict progression of *IDH*_MT glioma.

### Identification of genetic features that correlate with *IDH1* mutation status

**Figure 4:**
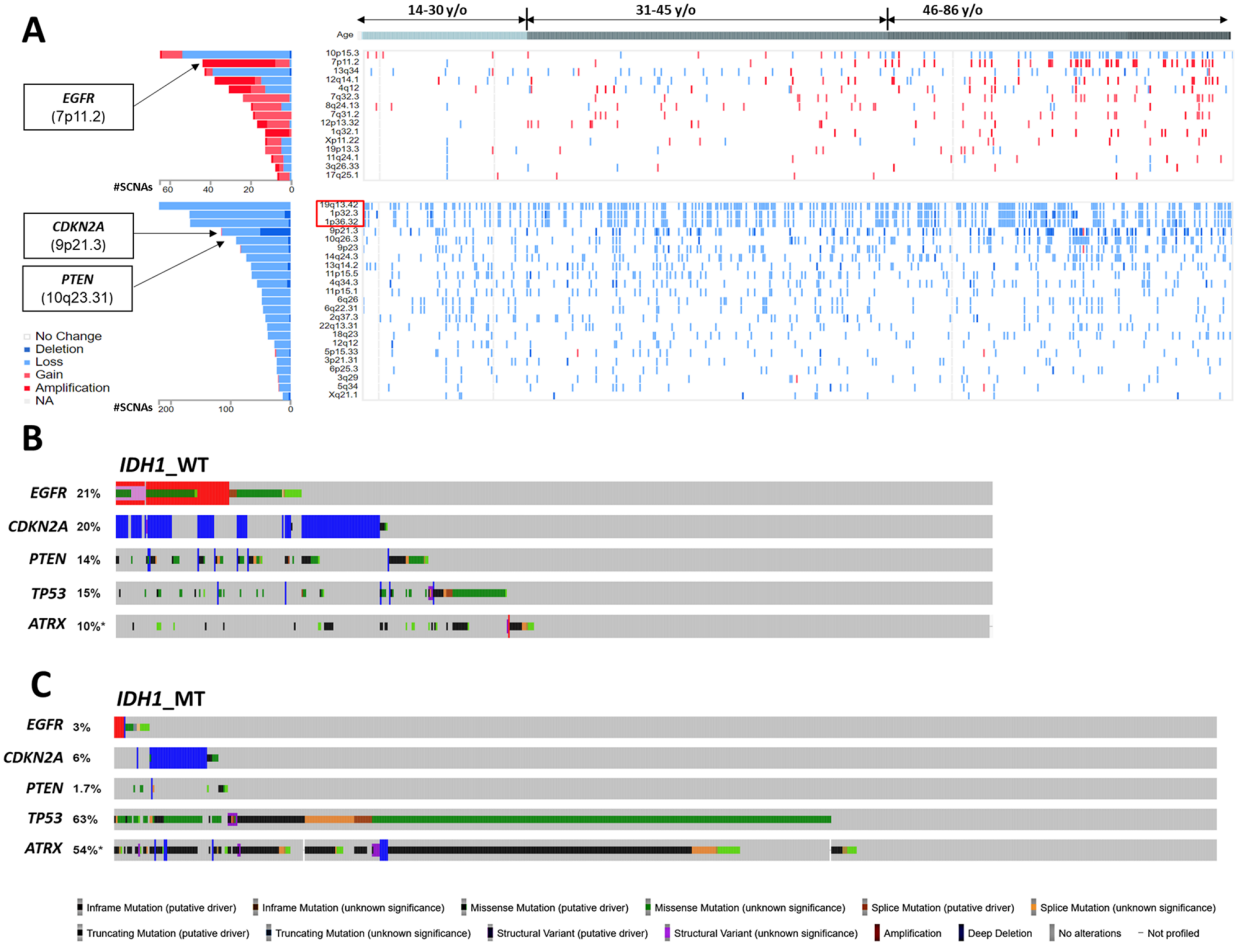
Identification of molecular markers as potential inputs for neural network construction. (**A**) WHO Grade 2 and 3 glioma cases from TCGA dataset were aligned based on age at diagnosis. Cases with copy number gain (top panel) or loss (lower panel) on cytobands that have most frequently copy number variations (CNVs) were color-coded. Genes selected as inputs in our final model of neural network were indicated. (**B**, **C**) 1309 WHO Grade 2 and 3 glioma patients of AACR GENIE v13.0 dataset were grouped into *IDH1* mutated (*IDH1*_MT) and *IDH1* wildtype (*IDH1*_WT) cohorts, and aligned based on CNVs status of *EGFR*, *CDKN2A*, and *PTEN*, as well as mutation status of *TP53* and *ATRX*. *NA* data not available, *SCNAs* number of somatic copy number alterations.

As copy number variations (CNVs) closely regulate glioma tumorigenesis, radioresistance and prognosis^15–18^, we further evaluated CNVs for patients diagnosed at different age groups. Individual WHO Grade 2 and 3 glioma patients from TCGA dataset (n = 516) were aligned by age at diagnosis (Fig. 4A). Most prevalent CNVs were identified in chromosome arms 7p, 9q, 10p, 10q, 19q and 1p (Fig. 4A). We found that cytobands 10p15, 9p21, 10q26, and 7p11.2 have the most prevalent and discrepant distribution among different age groups, with elderly glioma patients have more frequent 10p15, 9p21, 10q26 copy number (CN)-loss, and 7p11.2 CN-gain (Fig. 4A). Corresponding oncogenes located on those cytobands including *EGFR* (7p11.2), *CDKN2A* (9p21.3), and *PTEN* (10q23.31), were identified. 1,309 WHO Grade 2 and 3 glioma patients from AACR GENIE v13.0 datasets were grouped based on *IDH1* mutation status, and individual cases were aligned based on CNVs status of *EGFR*, *CDKN2A*, and *PTEN*, as well as mutation status of *TP53* and *ATRX* (Fig. 4B,C). Samples carrying *EGFR*, *CDKN2A*, *PTEN* CNVs tended to have wildtype *ATRX* and *TP53* in *IDH1*_WT cohort, whereas majority of samples with *EGFR*, *CDKN2A*, *PTEN* CNVs also carry *TP53* or *ATRX* mutation in the *IDH1*_MT cohort. These data indicates a different combination of *EGFR*, *CDKN2A*, *PTEN* CNVs, and *TP53*, *ATRX* mutation status reflect a unique genetic composition of glioma patients at different age groups and *IDH* mutation status.

### Model construction, dissection and calculation of GlioPredictor score

**Figure 5:**
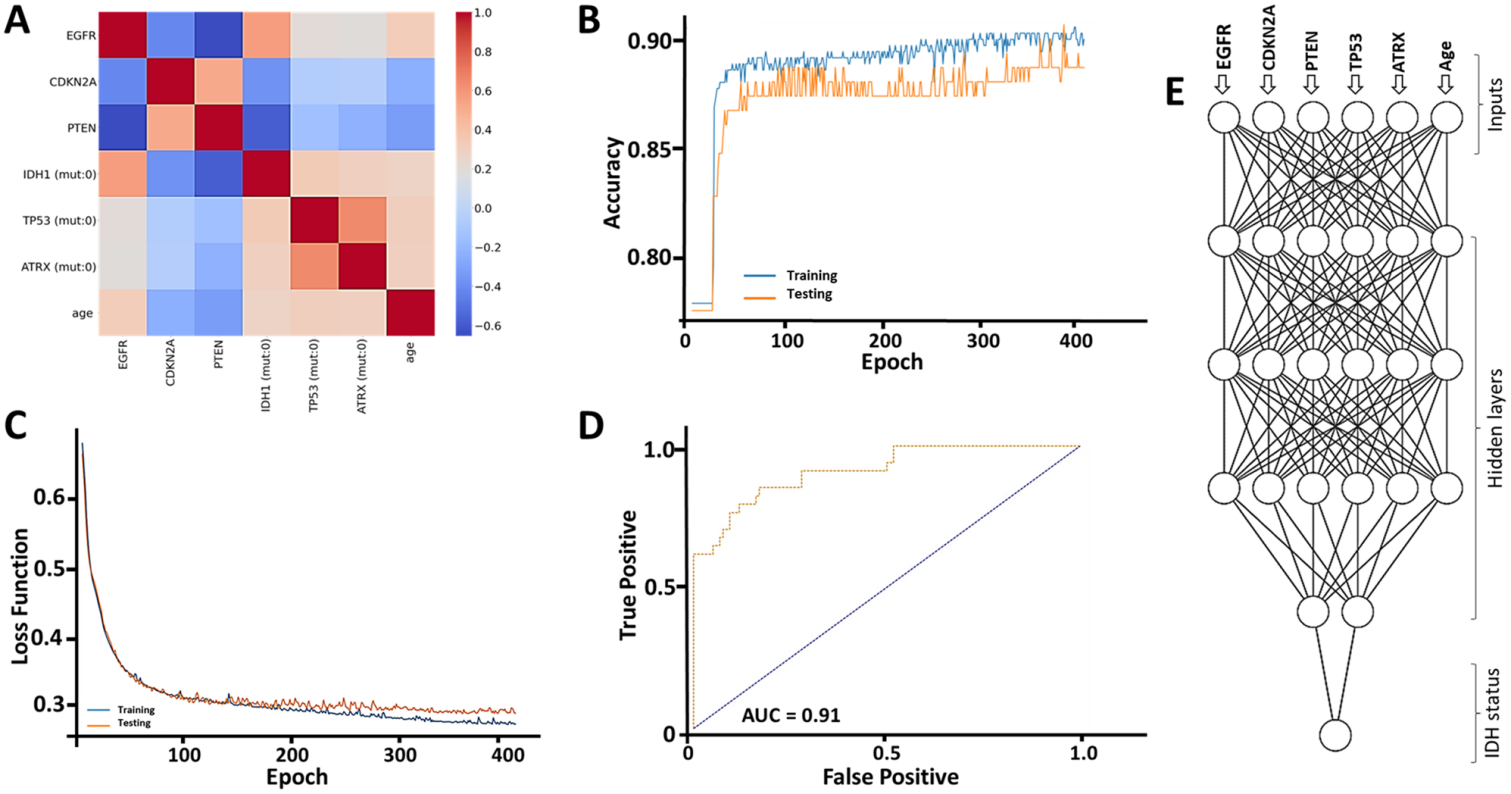
Artificial neural network (ANN) feature selection, target identification and ANN construction. (**A**) Correlation study of features of (CNVs of *EGFR*, *CDKN2A*, *PTEN*, and mutation status of *TP53, IDH1, ATRX,* and age at diagnosis). (**B**) Evaluation of prediction accuracy for both the test dataset and train dataset. (**C**) Evaluation of loss function for both the test dataset and train dataset. (**D**) ROC curve analysis of the built neural network model. (**E**) Schematic overview of the ANN model (6/6/6/6/2/1). Features selected as inputs are indicated.

The construction of artificial neural network (ANN) models requires multiple trials of different combination of inputs, and fine tuning of hyperparameters to achieve the best area under the receiver operating characteristic curve (AUC). Candidate inputs incorporated genetic information (mutation status of *TP53*, *ATRX, EGFR, TERT,* and *CIC*; CNVs of *EGFR*, *CDKN2A*, *PTEN*, *CUL2*), clinical parameters (i.e., age and gender), and histological features (i.e., astrocytoma, and oligodendroglioma). Variables with the strongest correlation with *IDH1* mutation status include CNVs of *EGFR* (r = 0.57), *PTEN* (r = − 0.58), *CDKN2A* (r = − 0.37), mutational status of *TP53* (r = 0.34) and *ATRX* (r = 0.31), and age at diagnosis (r = 0.28). We found that in our best performing ANN model, 6 inputs with predefined correlation (r > 0.2 or < − 0.2) can improve model performance (Fig. 5A). The best performing model had a structure of 6 inputs (mutation status of *TP53*, *ATRX,* CNVs of *EGFR*, *CDKN2A*, *PTEN*, and age at diagnosis) with 4 hidden layers (6/6/6/6/2/1). This was associated with prediction accuracy consistently around 90% in both the training set and validation set with AUC of 0.91 (Fig. 5B,D,E). No obvious overfitting was observed in the loss function analysis (Fig. 5C).

### Model evaluation

**Figure 6:**
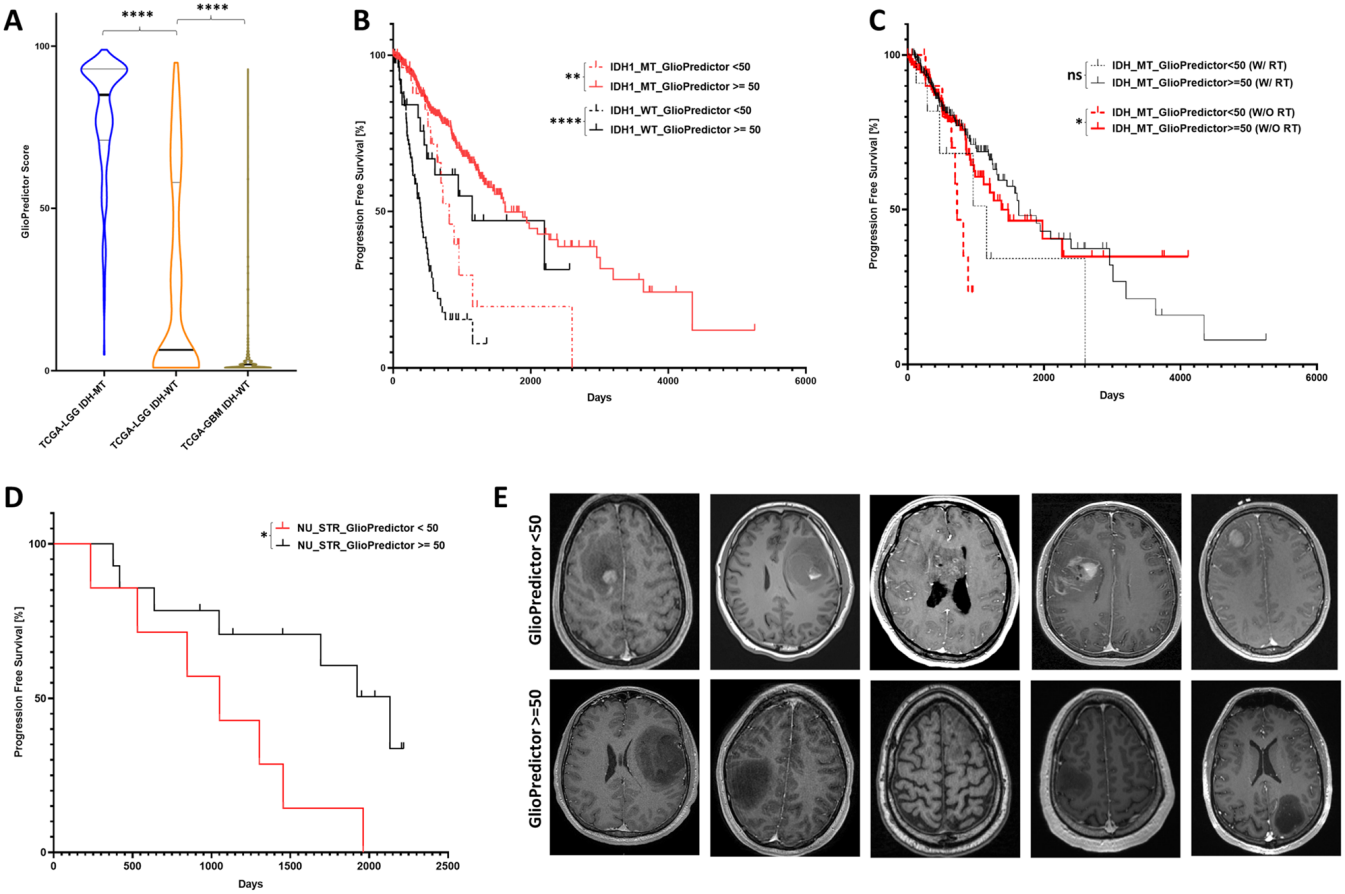
Performance assessment of GlioPredictor score in risk classification. (**A**) Violin analysis of GlioPredictor score in cohorts of WHO Grade 2 and 3 glioma TCGA dataset with mutated *IDH1* (TCGA IDH-MT), with wildtype *IDH1* (TCGA IDH-WT), and TCGA-Glioblastoma with wildtype *IDH1* (TCGA-GBM, IDH-WT). (**B**) The WHO Grade 2 and 3 glioma cases from TCGA dataset was subgrouped into cohorts of *IDH1*_MT (n = 286) and *IDH1*_WT (n = 112). PFS were evaluated for patients with GlioPredictor < 50 (n = 29) vs. those ≥ 50 (n = 362) (median PFS: 815 vs. 1629 days; *p* = 0.0019) for the *IDH1*_MT cohort, and GlioPredictor < 50 (n = 82) vs. those ≥ 50 (n = 30) (median PFS: 402 vs. 1147 days; *p* = 0.0007) for *IDH1*_WT cohort. (**C**) PFS analysis of the TCGA *IDH1*_MT cohort treated with adjuvant radiotherapy (w/RT, n = 211) and those without adjuvant RT (w/o RT, n = 147), which are further subgrouped based on their GlioPredictor score. (**D**) *IDH1*_MT Grade > 2 cases with subtotal resection (STR) derived from Northwestern University (NU) were subgrouped into GlioPredictor < 50 (n = 7) vs. those ≥ 50 (n = 14) (*p* = 0.0238). (**E**) Representative MRI of initial diagnostic MRI for NU_STR patients with GlioPredictor < 50 vs. those with GlioPredictor ≥ 50. *p < 0.05; **p < 0.01; ***p < 0.001; ****p < 0.0001.

The GlioPredictor score was calculated for glioma patients in the MSK and TCGA datasets. GlioPredictor score of 50 was used as a cutoff. Violin analysis for the distribution of GlioPredictor score in cohorts of WHO Grade 2 and 3 glioma patients in TCGA dataset with mutated *IDH1* (TCGA-*IDH1*_MT), with wildtype *IDH1* (TCGA-*IDH1*_WT), and TCGA-Glioblastoma with wildtype *IDH1* (TCGA-GBM *IDH1*_WT) showed significantly different pattern of distribution with mean GlioPredictor score of 79, 27, and 6, respectively (Fig. 6A, p < 0.001). Univariate analysis showed hazard ratio (HR) of 4.18 of GlioPredictor score < 50 in WHO Grade 2 and 3 glioma early progression (Table 1). Multivariate analysis on the construction set of the 6 inputs and GlioPredictor score showed GlioPredictor score < 50 (HR: 2.4, *p* = 0.001), older age (HR: 1.016, *p* = 0.008), *CDKN2A* CN-loss (HR: 0.67, *p* = 0.031), PTEN CN-loss (HR: 0.317, *p* = 0.0002), ATRX mutation (HR: 1.699, *p* = 0.024) were unfavorable prognostic factors for disease progression (Table 1).

**Table 1:** Univariate and multivariate Cox Regression analyses for progression free survival (PFS) for LGG patients with age and genetic alterations as covariables.

	Univariate analysis (PFS)			Multivariate analysis (PFS)		
	HR	95% CI of HR	*p* value	HR	95% CI of HR	*p* value
Age	1.029	1.018–1.041	< 0.001	1.016	1.004–1.029	0.008
EGFR	1.619	1.443–1.818	< 0.001	0.981	0.834–1.109	0.82
CDKN2A	1.7	0.941–3.068	0.079	0.67	0.467–0.963	0.031
PTEN	0.113	0.075–0.169	< 0.001	0.317	0.154–0.655	0.002
TP53	0.805	0.605–1.071	0.137	0.945	0.599–1.491	0.808
ATRX	0.905	0.676–1.211	0.5	1.699	1.071–2.695	0.024
GlioPredictor	4.188	3.068–5.716	< 0.001	2.417	1.426–4.096	0.001

### GlioPredictor in predicting glioma progression

**Figure 7:**
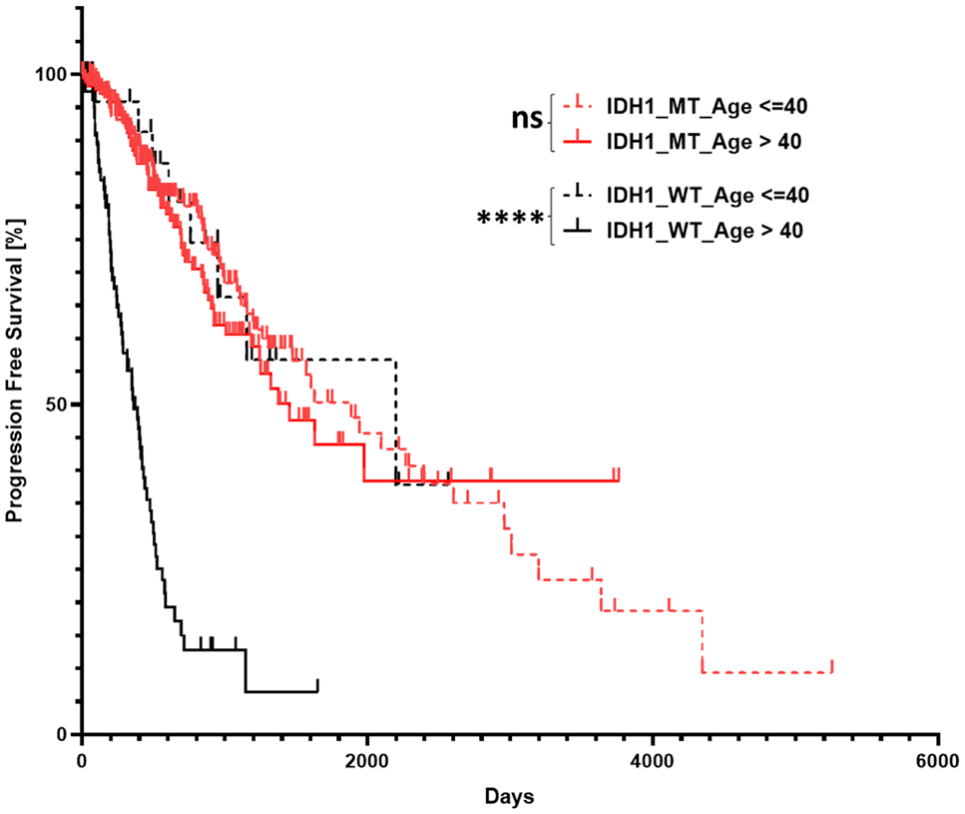
Evaluation of age at risk classification of TCGA dataset. WHO Grade 2 and 3 glioma patients from TCGA dataset was grouped into *IDH1*_ MT cohort based on age and analyzed for PFS with age > 40 (n = 176) vs. (age ≤ 40 (n = 215) (median PFS: 1886 vs. 1452 days;* p* = 0.459). TCGA dataset was then grouped into *IDH1*_ WT cohort based on age at diagnosis with age > 40 (n = 82) vs. age ≤ 40 (n = 30) (median PFS: 362 vs. 2197 days;* p* < 0.0001). ****p < 0.0001. *ns* non-statistically significant.

We then further attempted to test the efficacy of GlioPredictor score in predicting glioma progression. The WHO Grade 2 and 3 glioma patients in TCGA dataset were subgrouped into cohorts of *IDH1*_MT (n = 286) and *IDH1*_WT (n = 112). We found *IDH1*_MT glioma patients with GlioPredictor < 50 (n = 29) had significantly worse PFS than those with GlioPredictor ≥ 50 (n = 362) (median PFS: 815 vs. 1629 days; *p* = 0.0019; Fig. 6B), whereas no PFS difference were observed when *IDH1*_ MT patients were grouped based on age of age ≤ 40 vs. age > 40 (median PFS: 1886 vs. 1452 days; *p* = 0.459) (Fig. 7). Analysis of *IDH1*_MT with GlioPredictor < 50 (High Risk) vs. those GlioPredictor ≥ 50 (Low Risk) showed GlioPredictor High Risk patients have no obvious high risk clinicopathological features for early progression (Table 2). In the *IDH1*_WT cohort, again patients with GlioPredictor < 50 (n = 82) has significantly worse PFS than those with GlioPredictor ≥ 50 (n = 30) (median PFS: 402 vs. 1147 days; *p* = 0.0007; Fig. 6B). Interestingly, in TCGA *IDH1*_WT cohort, age over 40 (n = 82) was significantly associated with poor PFS than those age ≤ 40 (n = 30, median PFS: 362 vs. 2197 days; *p* < 0.0001; Fig. 7).

**Table 2:** Basic clinicopathological features of patients classified by GlioPredictor as High Risk (GlioPredictor < 50) vs. Low Risk (GlioPredictor ≥ 50).

	High risk	Low risk	*p* value
Histology (AS, %)	29	62	0.00208
Median age	37	39	ns
Grade 2 (%)	56	54	ns
Grade 3 (%)	44	46	ns

*ns* nonsignificant, *AS* Astrocytoma.

### GlioPredictor in prognosticating glioma treatment response

We further evaluated the potential of GlioPredictor in prognosticating adjuvant treatment response. In TCGA_*IDH1*_MT WHO Grade 2 and 3 glioma patients treated with adjuvant radiotherapy (w/RT, n = 211), no significant difference on PFS was observed between those with GlioPredictor < 50 (n = 11) vs. GlioPredictor ≥ 50 (n = 200, *p* = 0.1, Fig. 6C). For those without adjuvant RT (w/o RT, n = 147), we found statistically significant worse PFS for the cohort with GlioPredictor < 50 (n = 14) vs. GlioPredictor ≥ 50 (n = 133, *p* = 0.029, Fig. 6C), indicating adjuvant RT is warranted in this molecularly high-risk glioma cohort. We then studied the potential of GlioPredictor in prognostication of glioma patients who histologically would warrant adjuvant treatment. *IDH1*_MT WHO Grade 3 cases with subtotal resection (STR) were derived from Lurie Robert H. Lurie Comprehensive Cancer Center of Northwestern University (NU_STR) who had received adjuvant concurrent temozolomide and RT. We found a correlation among 87% of NU_STR patients with enhancing lesions on their initial post-surgical radiological imaging 87% with a GlioPredictor < 50 vs. 52% of patients with enhancement on radiological imaging with a GlioPredictor ≥ 50 as shown in Fig. 6E. For PFS analysis, NU_STR patients with Gliopredictor < 50 (n = 7) have earlier disease progression than those with GlioPredictor ≥ 50 (n = 14), *p* = 0.0238) (Fig. 6D).

## Discussion

This study provided a functional deep neural network (DNN) that can identify high-risk IDH-mutant glioma patients and assist with prognostication for post-operative management. The model we built first predicted *IDH1* mutation status in the TCGA dataset with a 90% accuracy and AUC score 0.91 with 6 readily available genetic and clinical characteristics including: *TP53* and *ATRX* mutation status, CNVs for *PTEN*, *EGFR,* and *CDKN2A*, and age at diagnosis. We then used the trained model to generate the GlioPredictor score, with a lower score reflecting a genetic background similar to *IDH1* wildtype. We then demonstrated that a low GlioPredictor score can identify a group of *IDH1*-mutant patient at higher risk of early progression. Therefore, GlioPredictor assessment is capable of integrating important molecular features and clinical information into a simplified risk stratification score.

Clinical trial results of Radiation Therapy Oncology Group (RTOG) 9802 and the European Organization for Research and Treatment of Cancer (EORTC) 22033–26033 suggests adjuvant radiotherapy (RT) either alone or in combination with chemotherapy for high-risk WHO Grade 2 glioma patients^13,14,19,20^. Prior to the molecular biomarker-based WHO 2021 classification, high-risk WHO Grade 2 glioma patients were often defined as patients with age > 40 years or a less than total gross resection, the criterion adopted from the RTOG 9802 trial and recommended in the most recent NCCN guidelines^13,14^. It is now known that risk assessment and corresponding treatment planning should incorporate a tumor’s genetic features as critical decision-making factors. However, molecular biomarkers have complex biological implications and are often interrelated; as such, a method to systematically access *IDH*-mutant glioma patients can add prognostic value.

The role of immediate adjuvant radiotherapy (RT) in *IDH*-mutant management is debatable, and concerns regarding RT-induced long-term neuropsychological side effects are not negligible^19,21,22^. All the molecular markers evaluated in this study, i.e., *TP53*, *ATRX*, *PTEN*, *EGFR,* and *CDKN2A,* have been proposed as radiosensitivity biomarkers of glioma. The GlioPredictor model integrates these markers with clinicopathologic information to provide a tool to evaluate the role of radiation therapy in glioma patients.

While we believe our model has good efficacy and applicability, several drawbacks remain that await further study. First and foremost, the sample size and tumor characteristics are limited based on features reported in public datasets. If more samples were available to train the neural network, we believe the performance will be further improved. Secondly, treatment-related details were not available for datasets involved in model training, validation, and cross validation. Thirdly, prospective studies are required to demonstrate the clinical applicability of the model, especially when the definition of glioma progression was not clearly specification in those public datasets. w Also, although we tested the trained model in several independent datasets, GlioPredictor was trained using TCGA dataset alone and therefore, sample bias and tumor heterogenicity may compromise the clinical applicability of the model. Last but not least, multiple clinical parameters such as size of the tumor, extent and anatomical location of the tumor involvement, extend of resection, neurological deficits, histology subtypes, gender, history of seizures, treatment received, patient baseline performance, as well as other biomarkers were not incorporated in the current version of GlioPredictor. Those parameters not included are critical for disease status evaluation and treatment recommendation, and can be potential cofounding factors of GlioPredictor score. Furthermore, the GlioPredictor model was not validated in paired recurrent tissues, paired progressed MRI brain. Utilization of GlioPredictor is not a replacement for those known risk assessment criteria. Instead, it is intended to facilitate comprehensive molecular assessment of glioma when clinical decision-making is increasingly dependent upon a panel of seemingly unrelated biomarkers ranging from copy number variation to mutations.

### Supplementary Information

Supplementary Figure 1.

## Data Availability

The datasets generated and/or analyzed during the current study are available in the cBioportal repository, https://www.cbioportal.org/. Northwestern University (NU) dataset (n = 21) is not publicly available due IRB restrictions (Study number STU00213078, August 2020). Please contact the corresponding authors (S.H.Z, T.O.T.) for request of access.

## References

[1] Lin Z, Yang R, Li K (2020). Establishment of age group classification for risk stratification in glioma patients. BMC Neurol..

[2] Oberheim Bush NA, Chang S (2016). Treatment strategies for low-grade glioma in adults. J. Oncol. Pract..

[3] Molinaro AM, Taylor JW, Wiencke JK (2019). Genetic and molecular epidemiology of adult diffuse glioma. Nat. Rev. Neurol..

[4] Franceschi E, Tosoni A, Bartolini S (2020). Histopathological grading affects survival in patients with idh-mutant grade ii and grade iii diffuse gliomas. Eur. J. Cancer.

[5] Mellinghoff, I. K. *et al*. INDIGO Trial Investigators. Vorasidenib in IDH1- or IDH2-Mutant Low-Grade Glioma. *N. Engl. J. Med*. **389**(7), 589–601. 10.1056/NEJMoa2304194 (2023). PMID: 37272516. 10.1056/NEJMoa2304194 PMC11445763 37272516

[6] Whitfield BT, Huse JT (2022). Classification of adult-type diffuse gliomas: Impact of the world health organization 2021 update. Brain Pathol..

[7] Louis DN, Perry A, Wesseling P (2021). The 2021 who classification of tumors of the central nervous system: A summary. Neuro Oncol..

[8] Krogh A (2008). What are artificial neural networks?. Nat. Biotechnol..

[9] Bau D, Zhu JY, Strobelt H (2020). Understanding the role of individual units in a deep neural network. Proc. Natl. Acad. Sci. USA.

[10] Zhang Y, Lin H, Yang Z (2019). Neural network-based approaches for biomedical relation classification: A review. J. Biomed. Inform.

[11] Renganathan V (2019). Overview of artificial neural network models in the biomedical domain. Bratisl. Lek. Listy..

[12] Vogelstein JT, Bridgeford EW, Tang M (2021). Supervised dimensionality reduction for big data. Nat. Commun..

[13] Bell EH, Zhang P, Shaw EG (2020). Comprehensive genomic analysis in nrg oncology/rtog 9802: A phase III trial of radiation versus radiation plus procarbazine, lomustine (ccnu), and vincristine in high-risk low-grade glioma. J. Clin. Oncol..

[14] Buckner JC, Shaw EG, Pugh SL (2016). Radiation plus procarbazine, ccnu, and vincristine in low-grade glioma. N. Engl. J. Med..

[15] Sanson M, Hosking FJ, Shete S (2011). Chromosome 7p11.2 (egfr) variation influences glioma risk. Hum. Mol. Genet..

[16] Yadav AK, Renfrow JJ, Scholtens DM (2009). Monosomy of chromosome 10 associated with dysregulation of epidermal growth factor signaling in glioblastomas. JAMA.

[17] Lopez GY, Van Ziffle J, Onodera C (2019). The genetic landscape of gliomas arising after therapeutic radiation. Acta Neuropathol..

[18] Brennan CW, Verhaak RG, McKenna A (2013). The somatic genomic landscape of glioblastoma. Cell.

[19] Baumert BG, Hegi ME, van den Bent MJ (2016). Temozolomide chemotherapy versus radiotherapy in high-risk low-grade glioma (eortc 22033–26033): A randomised, open-label, phase 3 intergroup study. Lancet Oncol..

[20] Halasz LM, Attia A, Bradfield L (2022). Radiation therapy for idh-mutant grade 2 and grade 3 diffuse glioma: An astro clinical practice guideline. Pract. Radiat. Oncol..

[21] Klein M, Drijver AJ, van den Bent MJ (2021). Memory in low-grade glioma patients treated with radiotherapy or temozolomide: A correlative analysis of eortc study 22033–26033. Neuro Oncol..

[22] Koutsarnakis C, Neromyliotis E, Komaitis S (2021). Effects of brain radiotherapy on cognitive performance in adult low-grade glioma patients: A systematic review. Radiother. Oncol..

