# Supplementary material for: GlioPredictor: a deep learning model for identification of high-risk adult IDH-mutant glioma towards adjuvant treatment planning
Source: Sci Rep. 2024 Jan 25;14:2126. doi: 10.1038/s41598-024-51765-6 (PMC10808248; doi:10.1038/s41598-024-51765-6)
Supplement: Supplementary file 1 — Supplementary Figure 1. [file 41598_2024_51765_MOESM1_ESM.docx]

**Supplementary**


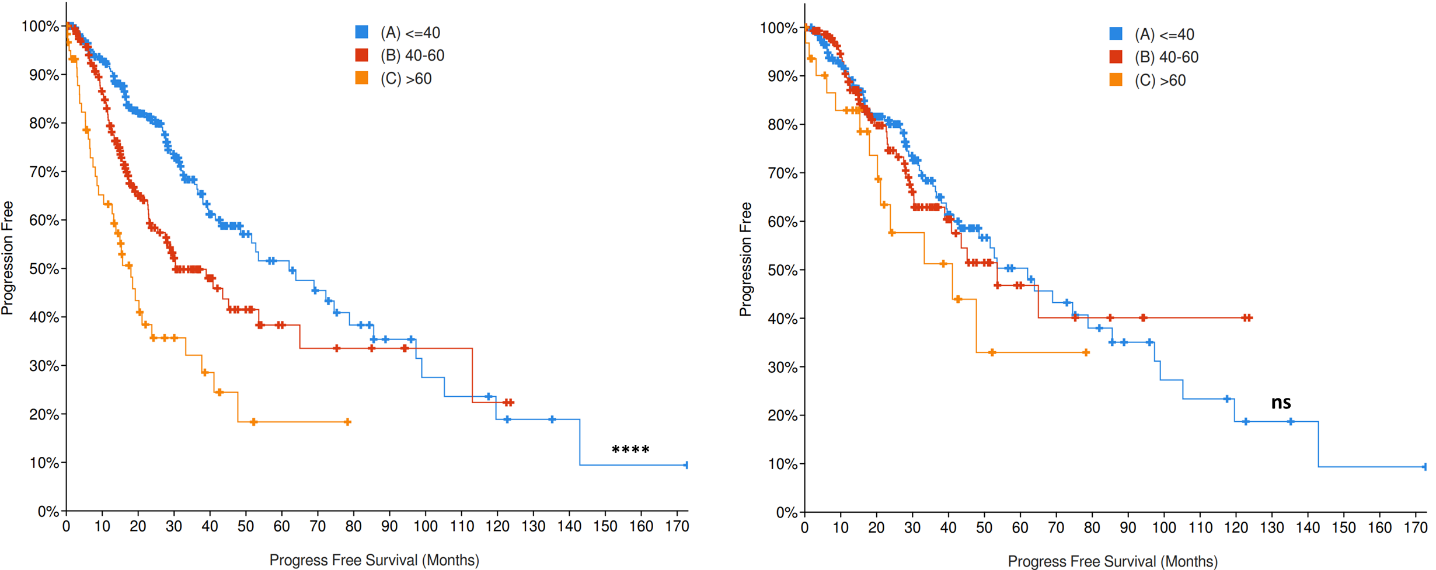


**Supplementary Figure 1. Evaluation of age as a risk factor in TCGA and TCGA *IDH1*_MT cohorts. A.** Progression free survival (PFS) of WHO Grade 2 and 3 glioma patients at different age groups of 18 to 40 (n = 254), 40 to 60 (n = 197), >60 (n = 61) from TCGA. **B.** PFS of TCGA *IDH1*_MT patients at different age groups of 18 to 40 (n = 218), 40 to 60 (n = 143), >60 (n = 32) from MSK dataset with primary samples available (*p*=0.228). ****: *p*<0.0001; ns: statistically nonsignificant.
